# *In Vitro* Biological Characterization of Silver-Doped Anodic Oxide Coating on Titanium

**DOI:** 10.3390/ma13194359

**Published:** 2020-09-30

**Authors:** Oleksandr Oleshko, Iryna Liubchak, Yevheniia Husak, Viktoriia Korniienko, Aziza Yusupova, Tetiana Oleshko, Rafal Banasiuk, Marek Szkodo, Igor Matros-Taranets, Alicja Kazek-Kęsik, Wojciech Simka, Maksym Pogorielov

**Affiliations:** 1 Biomedical Research Centre, Sumy State University, 40018 Sumy, Ukraine; oleshkosanya007@gmail.com (O.O.); irinalybchak@gmail.com (I.L.); evgenia.husak@gmail.com (Y.H.); vicorn77g@gmail.com (V.K.); ms.aziza.yusupova@gmail.com (A.Y.); t.oleshko@med.sumdu.edu.ua (T.O.); 2 NanoWave, 02-676 Warsaw, Poland; rafal.banasiuk@nanopure.pl; 3 Institute of Biotechnology and Molecular Medicine, 80-172 Gdańsk, Poland; 4 Mechanical Faculty, Gdańsk University of Technology, 80-233 Gdańsk, Poland; marek.szkodo@pg.edu.pl; 5 Dnipro Medical Institute of Traditional and Nontraditional Medicine, 49005 Dnipro, Ukraine; alusik255@gmail.com; 6 Faculty of Chemistry, Silesian University of Technology, 44-100 Gliwice, Poland; 7 NanoPrime, 39-200 Dębica, Poland

**Keywords:** plasma electrolytic oxidation, titanium, AgNPs, biocompatibility, antibacterial coatings

## Abstract

Despite the high biocompatibility and clinical effectiveness of Ti-based implants, surface functionalization (with complex osteointegrative/antibacterial strategies) is still required. To enhance the dental implant surface and to provide additional osteoinductive and antibacterial properties, plasma electrolytic oxidation of a pure Ti was performed using a nitrilotriacetic acid (NTA)-based Ag nanoparticles (AgNP)-loaded calcium–phosphate solution. Chemical and structural properties of the surface-modified titanium were assessed using scanning electron microscopy (SEM) with energy dispersive X-ray (EDX) and contact angle measurement. A bacterial adhesion test and cell culture biocompatibility with collagen production were performed to evaluate biological effectiveness of the Ti after the plasma electrolytic process. The NTA-based calcium–phosphate solution with Ag nanoparticles (AgNPs) can provide formation of a thick, porous plasma electrolytic oxidation (PEO) layer enriched in silver oxide. Voltage elevation leads to increased porosity and a hydrophilic nature of the newly formed ceramic coating. The silver-enriched PEO layer exhibits an effective antibacterial effect with high biocompatibility and increased collagen production that could be an effective complex strategy for dental and orthopedic implant development.

## 1. Introduction

Dental implants have been the most effective therapy for the replacement of dental elements in the treatment of total or partial edentulism since the 1960s after the first reports of Ti implant application [1]. A relatively high success rate (over 90%) was accompanied by early and late postoperative complications and eventually implant loss [2]. Postoperative infection plays an important role in implant loss even though optimal aseptic surgical practices are followed and modern antibiotic regimens are applied during surgery [3]. After implantation, the competition for metal substrate colonization between bacteria and cells predicts successful osteointegration. The lack of stability and microgaps with oral fluid flow at the bone–implant interface leads to bacterial infiltration and subsequent bone loss [4].

Thus, new multifunctional biomaterials with enhanced surface properties that can offer regeneration potential and protection against microorganisms have been intensively studied [5,6]. Dental and orthopedic implant masts meet the following criteria: proper biomechanical properties, cytocompatibility, osteoinductivity, and osteoconductivity [7]. Taking into account the increasing number of infectious complications, proper osteoinductive response and antibacterial properties are two major challenges that orthopedic devices need to simultaneously address.

To enhance dental implant surfaces, complex osteointegrative/antibacterial strategies have been developed with the aim of successful teeth replacement therapy [8]. The coating material with both antimicrobial activity and biocompatibility is important and leads to enhancement of the probability of implant success [9]. The following functionalization strategies have already been applied to increase antibacterial potential: drug-loaded surfaces [10], silver-implanted surfaces [11], polymer-functionalized surfaces [12], anodized/oxidized/ion-implanted surfaces [13,14], UV-activated surfaces [15], nanoscale surfaces [16], etc. A systematic review by J. Grischke et al. concluded that highly modified surfaces enhance antimicrobial activity compared to that of commercial, pure titanium [8], but some research proved the significant cell toxicity of these surfaces [17,18], which limits the clinical application of new surfaces. Despite biocompatibility and effectiveness, production simplicity and adequate final cost are required for mass-production implants. Due to developmental progress in nanoengineering, different nanoparticle compositions are actively being utilized to find appropriate coatings for dental and orthopedic implants [19].

Silver has demonstrated advanced antibacterial effects and has been effectively used since ancient times, but nanotechnologies could be an effective way to increase its antimicrobial potential and to decrease possible side effects. Ag ions and Ag nanoparticles (AgNPs) are already known as effective agents for coating incorporation that can overcome antibiotic resistance in gram-positive and gram-negative bacteria [20]. Some studies confirm that, compared to gram-negative bacteria, gram-positive bacteria are more resistant to AgNP treatment due to their relatively thicker cell walls and greater content of peptidoglycan [21,22]. Despite the high antibacterial effects of AgNPs, some studies demonstrated potential cell toxicity and osteointegration inhibition, which has limited AgNP application [23,24]. For incorporation of AgNPs into biocompatible coatings, optimizing the NP concentration and size/shape control are widely used ways to overcome AgNP toxicity [25].

Plasma electrolytic oxidation (PEO) is extensively used to provide high-quality functional coatings over metallic substrates [26]. It has been shown that the various components of the electrolyte can impact formation of the oxide layers and deposition of the desired components [27]. Different nanoparticles (NPs) have been incorporated into classical calcium–phosphate PEO solutions to provide functional antibacterial effects, but some research still demonstrates different toxicity levels [28]. It should be noted that AgNP incorporation via PEO can prevent the release of Ag ions into the bloodstream due to immobilization of AgNPs deep inside the oxide layer and can prevent expected toxic effects [29]. With the aim of obtaining functional coatings, different organic and inorganic chelating agents, such as ethylenediaminetetraacetic acid (EDTA), could be used for the PEO process [30]. Nitrilotriacetic acid (NTA) is a widely used chelating agent utilized for fluorescent agent development [31] and drug encapsulation [32]. NTA could be an effective agent in improving ceramic coating over the metal substrate using PEO technology with the addition of AgNPs.

The current research aimed to develop a functional coating in Ti-based alloys using an NTA-based AgNP-loaded calcium–phosphate solution and to evaluate the structural and chemical properties as well as the biocompatibility and antibacterial effectiveness.

## 2. Materials and Methods

### 2.1. Materials

Pure commercial Ti obtained from IWET (Kleosin, Poland) was prepared as 10-mm diameter cylindrical samples with a height of 4 mm for use in the experiment. All chemicals for PEO were purchased from Sigma Aldrich (Darmstadt, Germany) and used as received.

*Staphylococcus aureus* strain B 918, obtained from the National Collection of Microorganisms (D. K. Zabolotny Institute of Microbiology and Virology, Ukraine), was used in the experiment. All bacteriological media were purchased from (Mumbai, India), and Alamar blue was purchased from Invitrogen (CarIsbad, CA, USA). For the cell culture study, all media and reagents were purchased from Gibco^®^ (Waltham, MA, USA). U2OS cells were obtained from the collection of Sumy State University.

### 2.2. AgNP Synthesis

Silver nanoparticle (AgNP) synthesis was performed in a stainless-steel UltraViolet reactor (Nanowave, Warsaw, Poland). For every 200 mL of deionized water, 400 mg of polyvinyl pyrrolidone K30 and 120 mg of silver nitrate were added and vigorously mixed. After obtaining a homogenous mixture, 150 µL of 20% sodium hypochlorite was added. The reaction was performed under rigorous stirring for 1 h. In the next step, the obtained silver colloid was filtered using a reverse osmosis membrane (Osmotec M300-O) (Shapar, India) with pressure in the range between 2.8 and 3 bar until a silver concentration of 3 g/L was obtained. The nanoparticles were used as is without any further purification.

### 2.3. AgNP Characterization

To determine the size of the nanoparticles, their shape, and their chemical composition, tests were carried out using SEM (JEOL JSM-7800F) (Jeol Ltd., Akishima, Tokyo, Japan) equipped with an energy dispersive X-ray (EDX) spectroscopy analyzer. For this purpose, a sample made of chrome steel in the shape of a cylinder, 10 mm in diameter and 10 mm high, was prepared. Before microscopic examination, the flat surface of the sample was ground and polished to obtain an ultrasmooth surface. Then, a few drops of the colloidal mixture were applied to the polished surface of the sample and allowed to dry at 20 °C. Microscopic observations were made using an electron beam with an accelerating voltage of 5 kV using secondary electrons. An electron beam with a higher accelerating voltage of 15 kV was used to measure the chemical composition of the nanoparticles with the use of an EDX method.

### 2.4. Plasma Electrolytic Oxidation

The PEO process was performed using a DC power supply (PWR 800H, Kikusui, Japan) at limiting voltages of 250 and 300 V for 5 min (initial anodic current density = 150 mA/cm^2^). As the electrolyte, a suspension of Ag nanoparticles (180 mg/L) in a solution containing organic chelating agent (NTA), Ca(OH)_2_, and KH_2_PO_4_ was used [33]. Details of the PEO process were described in [34]. All samples were rinsed with distilled water and ultrasonically cleaned in deionized water and 2-propanol for 5 min prior to PEO treatment. To remove any unattached AgNPs, after the PEO process, the treated samples were ultrasonically cleaned in 2-propanol for 5 min and rinsed in deionized water. Next, the samples were dried on air.

### 2.5. Surface Characterization

After PEO, the surfaces were examined using a scanning electron microscope SEO-SEM Inspect S50-B (FEI, Brno, Czech Republic; accelerating voltage-15 kV). To assess the chemical composition of the surface of the samples, an energy-dispersive X-ray spectrometer (AZtecOne with X-MaxN20, Oxford Instruments plc, Abingdon, UK) accompanied by SEM was used. Before the SEM and EDX analyses, an ultrathin graphite film was sprayed onto the surface of all samples. SEM micrographs were examined with ImageJ 1.50c. software (https://imagej.nih.gov/ij/) (Version 1.5, University of Wisconsin, Madison, WI, USA) to define the average pore distribution. Spots for measurement were randomly chosen from three samples in each group (20 spots from each sample). The results are reported as the average value ± standard deviation.

The contact angle is frequently used to characterize the wettability of the surface. Contact angle (CA) measurements were made using a video-based optical contact angle measuring instrument (OCA 15 EC, Series GM-10-473 V-5.0, Data Physics, Filderstadt, Germany) and software (SCA_20U version 5.0.32, DataPhysics Instruments, Filderstadt, Germany). Ultrapure water droplets of approximately 0.2 μL were dropped onto the solid surface of the samples through a syringe at room temperature. At least five different regions of each surface were measured, and the average value was noted. The CA data were recorded for distillate water for at least three parallel samples.

### 2.6. Bacterial Adhesion test

Bacterial adhesion and inhibition properties of the samples were assessed against gram-positive bacteria. *Staphylococcus aureus* (S. aureus, strain B 918) was utilized as the representative strain of gram-positive bacteria in this assay. After culturing on solid medium for 24 h, inoculation of the strain was resuspended to a final density of 1 × 10^6^ colony-forming units (CFUs)/mL in liquid medium using McFarland standards.

The samples were sterilized by ultraviolet irradiation. Three groups of the samples (PEO-coated samples (TiP-250 and TiP-300), AgNP-loaded PEO-coated samples (TiP-250Ag and TiP-300Ag), and unreated Ti as a control (TiC) were incubated horizontally in static conditions with 2 mL of the bacterial suspension in a 24-well plate at 37 °C for 2, 4, 6, and 24 h.

The bactericidal mode for the samples was verified by an inhibition assay using the streak plate technique. Ten-microliter aliquots from each well were pipetted onto nutrient agar at each time point of the experiment. CFUs/mL were calculated by counting the visible colonies.

Next, the samples were removed with sterile forceps and gently washed in a fresh 24-well plate with 2.0 mL of sterile phosphate buffer (PBS) (pH 7.2) three times to remove loosely adhered bacteria. Then, the discs were placed in sterile tubes with 1.0 mL of PBS and the adherent bacteria on the samples were dislodged by ultrasonication for 1 min in an ultrasonic bath (B3500S-MT, Bransone Ultrasonics Co., Shanghai, China) followed by vortexing (Mini Rocker-Shaker, BioSan MR-1, Riga, Latvia) at maximum power for 1 min to remove bacteria adhered to the surface [31]. The number of CFUs on the solid medium was counted after 24 h of cultivation of 10 µL aliquots of PBS from the sonicated tubes using the streak plate technique. All experiments were performed in triplicate and are displayed logarithmically.

### 2.7. Cell Culture Biocompatibility Test

The U2OS cell line growing in a standard environment (DMEM/F-12 media under standard culture conditions of humidified air containing 5% CO_2_ at 37 °C) was used in the experiment. Alloy samples were sterilized in an autoclave, and each sample was placed in a separate well of a 24-well cell culture plate and immersed in DMEM overnight. On the next day, the medium was removed and U2OS cells were seeded on each sample and in the wells without samples (as positive control) at a cell density of 10^4^ cells per well. Cell adhesion at 24 h and cell proliferation on samples were assessed by a Alamar blue colorimetric assay as previously described [34]. As a negative control, Alamar blue solution was added to the wells containing only culture medium without cells. As a positive control, Alamar blue solution was added to the wells that contained only cells without samples (tissue culture plastic (TCP) control). The cells were quantified at different time intervals: 1, 3, and 7 d. All experiments were repeated 3 times.

### 2.8. Collagen Deposition Assay

Collagen, which was synthesized by U2OS cells and accumulated on samples, was detected through staining with Sirius Red dye. The staining was performed as follows [35]. U2OS cells were seeded on each sample at a cell density of 10^4^ cells per well, and on the 7th and 14th days of incubation, samples were transferred to another 24-well plate and washed 3 times with ice-cold PBS (4 °C). Then, 1.5 mL of Bouin’s solution was added to each well for 1 h at room temperature. After the solution was removed, samples were rinsed with cold tap water and dried in a fume hood overnight. On the next day, 1.5 mL of Sirius Red dye was added to the samples for 1 h and then removed and each well was washed 4 times with 0.01 M HCl. One milliliter of 0.1 M NaOH solution was added to each well to recover the bound dye. The plate was placed on a shaker for 30 min, after which 100 µL of eluted dye from each well was transferred to a 96-well plate and the absorbance was measured using a Multiskan FC (Thermo Fisher Scientific, Waltham, MA, USA) plate reader at a wavelength of 570 nm.

### 2.9. Statistics

The results are expressed as the mean ± standard deviation. One-way analysis of variance using GraphPad Prism 8.0 (Version 8.0.2, GraphPad Software, San Diego, CA, USA) was used to determine the level of significance (*p* < 0.05 was defined as significant).

## 3. Results

### 3.1. Structural and Chemical Composition of AgNPs and PEO Coating

**Figure 1:**
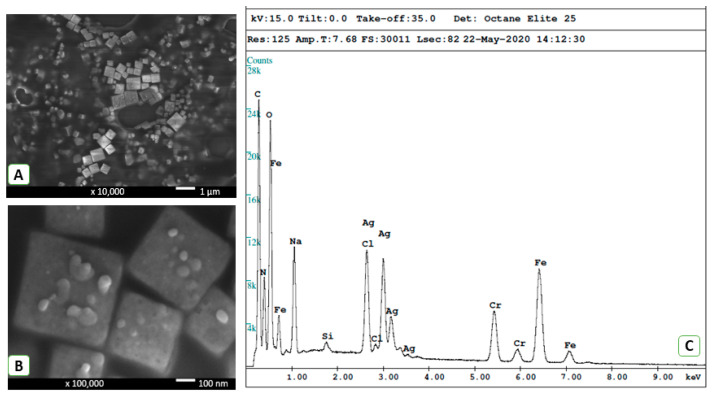
Scanning electron microscopy images of silver nanoparticles at 10,000× (**A**) and 100,000× (**B**) magnification coupled with the EDX pattern (**C**) obtained for a colloidal mixture.

Before PEO of the Ti alloy, we conducted detailed characterization of freshly prepare AgNPs. As shown in Figure 1, the synthesis resulted in silver nanoparticles in the shape of cubes with dimensions in the range from 80 nm to 800 nm. Small silver nanoparticles with dimensions on the order of several dozen nm and spherical shapes could also be observed on the cube walls (Figure 1B). The membrane concentration process may have caused formation of these smaller nanoparticles. The EDX spectra (Figure 1C) show the presence of silver. Carbon, oxygen, and nitrogen atoms originate from polyvinyl pyrrolidone (end-capping agent). The sodium chloride crystals are present by the conversion of sodium hypochlorite to sodium chloride during synthesis. The signals of chromium and iron occur due to the sample-carrying disk utilized during the measurement.

**Figure 2:**
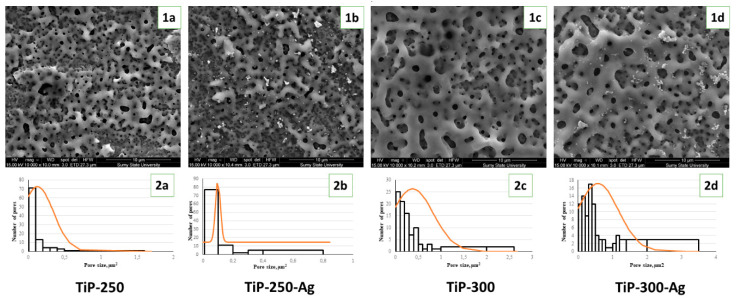
Scanning electron microscopy images (**1a**–**1d**) and pore size distribution (**2a**–**2d**) after plasma electrolytic oxidation (PEO) of Ti implants in nitrilotriacetic acid (NTA)-containing solutions.

PEO of pure Ti in the NTA-containing solution provided formation of a rough, thick oxide layer with a highly porous structure (Figure 2(1a–1d)). The surface treated with 250 V provided formation of mesoporous structures with pore sizes ranging from 300 nm to 2 µm. The nanopores grouped in dense areas separated with spots in micrometer dimension pores. The addition of AgNPs slightly decreased the surface porosity from 0.127 ± 0.22 µm^2^ to 0.1 ± 0.017 µm^2^, and nanoparticle aggregates were present over the ceramic coating. Increasing the voltage during the PEO process led to significant porosity growth both in pure (0.375 ± 0.45 µm^2^) and AgNP-loaded solutions (0.572 ± 0.6 µm^2^). It should be noted that pore size significantly increased and nanosized pores were distributed more evenly at the application voltage higher than 250 V mode. Histograms of the pore size distribution confirm the formation of micropores at 300 V and a more uniform size distribution after AgNP addition (Figure 2(2a–2d)).

EDX analysis showed the formation of a Ca–P ceramic coating with Ca/P ratios from 1.1 in the TiP-250-Ag sample to 1.7 in the TiP-300-Ag sample (Table 1). The high voltage led to a significant increase in the Ca/P ratio that probably influenced cell attachment and proliferation. The silver was present after treatment at both voltages, but at 300 V application voltage, the Ag concentration grew two-fold.

**Figure 3:**
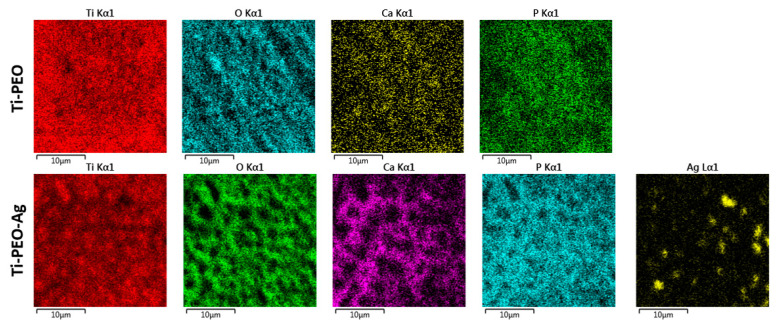
Exemplary EDX mapping of element distribution on the PEO layer in NTA-containing solution (upper row) and after Ag nanoparticles (AgNP) doping (lower row): images represent 300 V mode.

EDX mapping confirmed the uniform distribution of O, Ca, and P in the ceramic coating. The AgNPs incorporated into the PEO coating formed aggregates that were visualized as bright spots (Figure 3). There were no significant differences in the element distributions between the 250 V and 300 V modes. These results may suggest that the main difference between the PEO parameters is in the ceramic layer morphology and that the chemical compositions of both modes are the same (Table 2).

### 3.2. In Vitro Investigations

#### 3.2.1. Cell Proliferation and Collagen Production Assays

**Figure 4:**
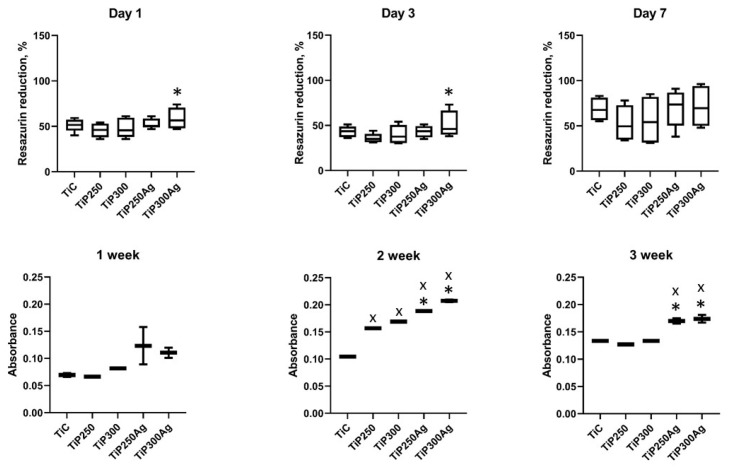
U2OS cell viability during 7 days of cocultivation over the Ti samples (upper row) and collagen production assay during 3 weeks of cell cultivation (bottom row): ^x^ significant difference with control group; * significant difference with Ag-free group.

Cell attachment and proliferation over 7 days demonstrated the absence of cell toxicity and proved the high biocompatibility of both pure Ti and PEO-coated implants (Figure 4, upper row). There was a significant increase in cell attachment on the Ti sample treated with Ag nanoparticles on the 1st day of the experiment compared to that of samples from the Ag-free group in 300 V mode, with a similar trend on the 3rd day of the experiment. By the end of the experimental period, the cells remained viable on all samples with no significant difference between groups.

There was no significant difference in the levels of collagen produced by cells during the 7-day period (Figure 4, low row). In the 2nd week of the experiment, we observed an increase in collagen production in both PEO-coated samples. Moreover, there was a significant increase in the levels of collagen synthesized on disks treated with Ag nanoparticles. It is important to note that the mode of PEO (250 or 300 V) did not contribute to cell proliferation and collagen synthesis. In the 3rd week, the levels of collagen detected in the samples were lower than those in the 2nd week. This can be explained by U2OS cell death due to lack of space on the sample surface because of active cell proliferation between 2 and 3 weeks of the experiment. Nevertheless, we observed a similar trend of increased levels of collagen on the surface of Ag-treated samples.

#### 3.2.2. Bacterial Adhesion and Inhibition Properties

**Figure 5:**
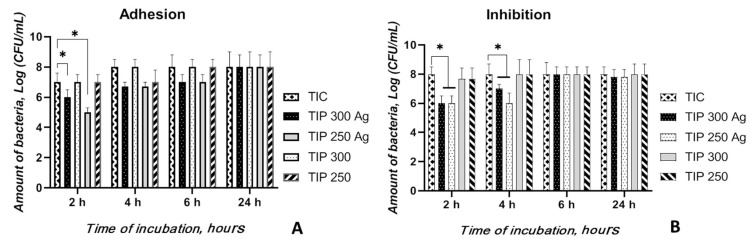
The number of viable bacteria adhered on the sample surfaces at different time points of the experiment (**A**) and the antibacterial activity of the samples by the inhibition assay (**B**). * A significant difference among the samples (*p* < 0.05).

As shown in Figure 5A, the untreated Ti alloy (TIC) was covered with bacterial colonies (the number of viable bacteria that adhered to the surfaces was 8 starting at the 4-h time point after logarithmic transportation), indicating that untreated Ti surfaces do not possess anti-adherent properties. PEO Ti (TiP-300 and -250) and AgNP-PEO Ti (TiP-300 Ag and -250 Ag) could inhibit bacterial adhesion and surface biofilm formation by Staphylococci at the early time points of the assay. Nevertheless, both the 300 and 250 TiP Ag samples inhibited a higher number of bacterial colonies than both TiP samples and the TIC alloy at 2, 4, and 6 h. However, the number of living bacteria on the surface of the TiP Ag samples was significantly lower than on the TIC alloy (*p* < 0.05) and TiP samples (*p* < 0.05) only after 2 h incubation, and no difference was observed among these of specimens at the 24-h time point.

Similar to the bacterial adhesion results, no difference was observed between TiP samples and TiC samples at the three time points (Figure 5B). The bacterial cell amounts after cocultivation with TIC Ag were lower than those for both TiP samples and TIC alloys at the 2-h and 4-h time points (*p* < 0.05) and compared to the untreated control at the 2-h time point (*p* < 0.05).

It can be suggested that the TiP Ag samples inhibited bacterial adhesion and eradicated a planktonic bacterial population with significantly superior performance due to Ag ion release from the coating. Decreasing the initial (during the initial 6-h period) bacterial adhesion onto biomaterial surfaces following implantation is essential to prevent implant-related infections.

## 4. Discussion

AgNP size and shape can significantly influence their antibacterial properties and cell toxicity. In our study, cubic AgNPs with dimensions ranging from 80 nm to 800 nm were used for metal surface doping during the PEO coating process. It has been demonstrated that AgNPs with a size of 10 nm exhibit moderate cell toxicity [36] in contrast to larger dimensions that did not alter cell viability [37,38]. Our data showed that the relatively large size of the freshly synthesized AgNPs demonstrated a high antibacterial effect with very low toxicity in both the pure Ti and with a PEO coating. In support of our data, there are many studies that demonstrated high antibacterial activities of AgNPs with sizes from 5 nm to 900 nm [39,40].

On the basis of the obtained results analysis and previous investigations, we can conclude that, after the PEO process, an oxide layer composed of titanium oxide and calcium phosphates was formed [41]. In the case of process conducted in the AgNP electrolytes, the oxide layer was enriched in silver, which was in the form of oxide [28,42,43]. Numerous data demonstrate the antibacterial effect of AgNPs incorporated into PEO coatings, but in most studies, AgNP use decreased the biocompatibility of implants [24,28]. The principal idea of the current research was loading of the implant surface with an antibacterial agent (AgNPs) to kill planktonic bacteria in implant tissue and to prevent bacterial adhesion and biofilm development. Completely formed biofilm is tremendously robust to elimination and can be the reason for antibiotic resistance by reducing antibiotic penetration across the extracellular matrix, providing bacterial cell differentiation and specialization to advance protection via planktonic population support [44].

Our study demonstrates that the PEO layer made from the Ca–P–NTA solution provides an appropriate environment for osteogenic cell growth and proliferation and exhibits antibacterial activity—both in terms of microorganism adhesion and proliferation. It should be noted that all PEO-coated samples demonstrated a significant elevation in collagen production, which is a positive prediction for bone tissue development [45]. A highly porous surface with Ca–P motifs should be an ideal environment for bone cell growth and enzymatic activity. Hulbert S. et al. demonstrated that osteons require mini pores in which the diameters range from 150 to 200 µm [46]. Later, Babuska V. found that lamellar bone and bone remodeling were highly favored by 200-µm pores created by a laser compared to 10–25-µm pores [47]. However, Stangl R et al. conducted histological analysis and determined that the optimal implant surface showed wavy structures with an average wavelength of 11.6 µm and with deviations in height of 1.4 µm [48]. Our data demonstrate that the pore size increases with voltage elevation during the PEO process, which significantly influences the cell viability and collagen production rate.

Another factor that stimulates cell adhesion and proliferation is surface hydrophilicity, which has been demonstrated in numerous studies [6]. The current study showed that new PEO coatings demonstrate an exceptional hydrophilic nature that increases with voltage elevation. Collagen synthesis also increased on the AgNP-doped surface, demonstrating possible stimulation of protein synthesis in osteogenic cells. Some studies have demonstrated the influence of Ag on collagen production *in vitro* but not on metallic substrates [49].

Our data demonstrate moderate antibacterial activities, especially focused on microorganism adhesion properties, of the developed AgNP PEO coating. It has been demonstrated that AgNPs incorporated into PEO coatings are able to release Ag ions during long-term exploitation [50]. The relatively uniform distribution of AgNPs within the PEO coating, demonstrated in EDX mapping, could provide slow ion release and effective antibacterial protection. The simultaneous antibacterial and biocompatible effects of the PEO coating open a new perspective for multifunctional implant development.

## 5. Conclusions

NTA-based calcium–phosphate solutions with AgNPs can provide formation of a thick, porous PEO layer in pure Ti implants. Voltage elevation leads to an increase in porosity and an increase in the hydrophilic nature of the newly formed ceramic coating. The silver-doped PEO layer provides an effective antibacterial effect with high biocompatibility and increased collagen production, which could be an effective complex strategy for dental and orthopedic implant development.

## Figures and Tables

**Table 1:** The semiquantitative EDX analysis results, wt.%.

Sample	Ti	O	C	Ca	P	Ag
**TiP-250**	52.6	12.3	5.2	16.8	13.1	-
**TiP-250-Ag**	49.2	9.8	7.2	17.6	15.9	0.3
**TiP-300**	57.3	7.5	4.0	19.7	11.5	-
**TiP-300-Ag**	54.8	7.9	6.1	18.3	12.2	0.7

**Table 2:** The results of contact angle measurement.

Parameter	TiC	TiP-250	TiP-250-Ag	TiP-300	TiP-300-Ag
**Contact angle (°)**	97.3 ± 5.8	54.9 ± 6.3 *	58.5 ± 3.6 *	34.2 ± 4.0 *^£^	31.9 ± 5.1 *^£^

* Significantly different from the control group, ^£^ significantly different from the 250 V group.
